# The Double-Edged Sword of Third-Party Resources: Examining Use and Financial Burden of Extracurricular Tools in Medical Students

**DOI:** 10.12688/mep.20120.2

**Published:** 2025-01-02

**Authors:** Saaniya Farhan, Drake Kienzle, Meryem Guler, Faizaan Siddique, Andres Fernandez, Dimitrios Papanagnou

**Affiliations:** 1 Sidney Kimmel Medical College at Thomas Jefferson University, Philadelphia, Pennsylvania, USA; 2 Conestoga High School, Berwyn, Pennsylvania, USA

**Keywords:** third-party resources, study aids, undergraduate medical education

## Abstract

**Background:**

According to the Association of American Medical Colleges (AAMC) Year 2 questionnaire, the percentage of students using online medical education videos (Boards and Beyond®Sketchy Medical®, Youtube) at least once per week increased from 47.7% (2015) to 70.1% (2022). Transition to virtual learning in 2020 fostered a greater reliance on these online third-party resources (i.e., educational tools distinct from formal medical curriculum), yet existing literature have rarely evaluated their efficacy. As students in this landscape, we aimed to review the usage, efficacy, and drawbacks of third-party resources and their financial burden on students.

**Methods:**

Four authors searched PubMed from June-July 2023 using the search terms “prevalence,” “efficacy,” and “disparities introduced by third-party board preparation resources.” All peer-reviewed articles in English were evaluated. No inclusion or exclusion criteria were implemented. References were mined for additional results, per the discretion of each reviewer.

**Results:**

31 studies were examined for common themes and findings to provide a snapshot of the existing literature. Studies suggest third-party resources are used in a task-dependent manner with a supplemental role to lectures during the preclinical years and a primary role for United States Medical Licensing Examination (USMLE) preparation during clinical years. Medical students access these resources to perform well on board exams, prompted by studies demonstrating their efficacy in increasing USMLE Step 1 scores. Though certain resources have been frequently cited for improving board performance (e.g., First Aid, UWorld), students combine multiple third-party resources to best serve their preparation. Subscription-pricing of most third-party resources and 12-month access prices range from $100 to $479.

**Conclusion:**

Third-party resource use may contribute to an increased financial strain on students. This, coupled with overwhelming medical student debt, may exacerbate socioeconomic disparities in medical education. Institutions should evaluate third-party resource use among students and consider provisions to increase access to them.

## Background

The last three decades have witnessed exponential growth of third-party extracurricular resources to supplement student training in medical school. Prior to the existence of online third-party resources (e.g., UWorld®, AMBOSS®, Sketchy Medical®, Boards & Beyond®), students reported using print commercial guides, official resources, and textbooks in preparation for board examinations ^1, 2^. However, with the advent of online tools, medical students have enhanced access to an exponentially increasing number of available resources ^3^. While some resources have some longevity, with First Aid by USMLERx™ celebrating its 30 ^th^ anniversary in 2019, many online resources (e.g., Pathoma®, Boards & Beyond®, Osmosis®, Sketchy Medical®, Pixorize®) became available within the last decade. The increasing variety in third-party resources enables students to use the most effective source of knowledge acquisition for their own personal needs ^4– 6^. However, these resources are costly and have the potential to widen inequities in student preparation and training ^4, 7^. Further, medical schools provide little curation of these resources along with a lack of guidance on their judicial use and how to incorporate them in students’ study plans to complement the institution’s curricular structure ^8^.

This expanding supply of third-party resources may arise from an increased demand from medical students, which was amplified by the COVID-19 pandemic. In a recent systematic review by Hirumi *et al.*, ^4^ of the 19 included studies found over 90% of students used third-party resources prior to the pandemic ^4^. Indeed, the transition to virtual learning during the COVID-19 pandemic contributed to a rise in use of third-party resources ^9^. As reported by Johansen *et al.*, results from the 2022 Association of American Medical Colleges (AAMC) Year 2 Questionnaire show that 22.4% more students used online medical education videos (e.g., Youtube™, Boards & Beyond®, Sketchy Medical®) at least once a week in 2022 (70.1%) versus in 2015 (47.7%) ^9^. Similarly, this research group found 75.8% of allopathic medical students at a community-based institution in 2021 said >50% of their learning came from resources outside course lectures, and 61% reported using more outside resources with the transition to virtual learning ^9^.

Given the ubiquitous use of third-party resources, more research is needed to consider the motivations and implications for medical students using these tools outside of their institution’s structured medical education program. Prior research has encouraged an evaluation of the benefits (i.e., efficacy) and drawbacks (i.e., financial burden) introduced by third-party resources ^4, 10, 11^. In this scholarly perspective, we explore the landscape of third-party resources and lay ground for exploratory research questions that could help address downstream inequities in their use in undergraduate medical education (UME).

The purpose of this review is to evaluate the implications of third-party resource use in UME. We hypothesize that access to third-party resources provides students with beneficial results (e.g., better board exam scores), perpetuating a reliance on these resources throughout medical training. This reliance creates an imbalance for students who cannot afford purchasing and/or subscribing to these ancillary resources, which adds to the existing barriers to equitable medical education.

## Literature search

The authors acknowledge that this is not a systematic or scoping review. Rather, the goal of this perspective paper was to perform a high-level review of the literature that would inform hypothesis generation for a more robust, subsequent review of the literature. This study was based upon the assumption that preclinical students commonly use third-party resources in medical school. As second-year medical students ourselves, we wanted to explore the literature to inform preliminary research questions for subsequent studies. As we later describe, xext steps will involve a scoping review to assess the financial implications and disparities perpetuated by the use of third-party resources outside of the traditional medical school curriculum.

We queried the PubMed database for peer-reviewed articles using the search terms “prevalence,” “efficacy,” and “disparities introduced by third-party board preparation resources” From June to July 2023. No inclusion or exclusion criteria were implemented besides only reviewing articles in English. Some references were mined for additional results as per the discretion of each reviewer. Four authors reviewed the 31 results and independently extracted the data for each article. Each study was appraised for key endpoints and conclusions, methods used to reach the initial hypothesis, strengths, limitations, and future directions suggested by the article. Studies were then examined for common ideas, topics, and findings that were repeatedly discussed, and themes were developed to capture patterns of meaning as a first pass of examining the literature on third-party resources. After common themes were collected, key findings were organized as questions to present observed findings and trends: “What types of third-party resources are used by medical students?”, “How do medical students incorporate third-party resources in their education?”, “What motivates medical students to use third-party resources?”, “Potential benefits of third-party resource use: are they effective for board preparation?”, “Potential drawbacks of third-party resource use: do they increase inequity in access to medical education?”.

## Results

### What types of third-party resources are used by medical students?

Medical students seek out and use a variety of third-party resources that aid their goals, regardless of the quantity ^4, 10, 12, 13^. For instance, researchers at the Kirk Kerkorian School of Medicine conducted post-exam surveys on medical students taking National Board of Medical Education (NBME®)-based exams bi-weekly and found that more than half of respondents (55%) used between six and eight resources for exam preparation ^10^. Among the most used resources were Boards & Beyond® (video lectures with post-video quizzes), First Aid™ (exam review book), and Kaplan® (practice question bank) in descending order ^10^. This may be partly explained by students’ desire to seek out third-party resources that are concise, easy to use, and that support long-term retention and recall ^4, 8, 14^.

Additionally, Hirumi *et al.* discusses a strong preference by medical students for resources that offer practice questions (e.g., Boards & Beyond®, Kaplan®, UWorld®, USMLE Rx by First Aid™, Sketchy Medical®) as they allow students to gain comfort with a format similar to that of the national board examinations ^4^. Such question banks (or Qbanks) provide an opportunity to practice well-supported educational techniques like spaced retrieval and active recall ^4, 15, 16^. Indeed, students also seek resources that cover “high-yield” topics, or content that has been regularly tested by board examiners and is considered essential content to review before examinations ^14, 17^. This may further motivate students to use third-party Qbanks, as practice questions generally emphasize “high-yield” content ^4, 18, 19^.

### How do medical students incorporate third-party resources in their education?

Studies suggest that third-party resources are used in a task-oriented manner, which seems to fluctuate based on educational phase. For instance, during the preclinical phase, where coursework is more classroom-based, students generally use third-party resources in a supplementary role while focusing on the in-house medical curriculum ^4, 8, 14, 17, 20^. Finn *et al.* found that, among first-year medical students surveyed, the most commonly used resources were lecture handouts and video recordings of lectures, with 89.8% and 87% of first-year students reporting regular use of them, respectively ^20^. Third-party resource use was also found to be prevalent among these first-year students, with over 50% indicating additional regular use of Anki™, Osmosis®, and Sketchy Medical® ^20^. This pattern of supplementation of formal curricula with third-party resources was similarly noted by Graff and colleagues ^8^. In their study, 70% of the first- and second-year students preferred lecture material as their primary learning resource, and over two-thirds of all students surveyed (of which 72.3% were self-designated as first- or second year) supplemented lecture material with at least one third-party resource every day or every other day ^8^. This phenomenon has been referred to as a “self-directed parallel curriculum,” in which students not only enhance their understanding of preclinical material with third-party resources but also begin their preparation for board examinations like the United States Medical Licensing Examination (USMLE®) Step 1 prior to dedicated study periods ^5, 8^. Such early preparation is particularly important, as numerous studies have marked it to be a significant predictor of improved Step 1 performance ^5, 18, 21, 22^. As students exit the preclinical phase and enter dedicated USMLE® Step 1 study periods, they modify their use of third-party resources accordingly. In contrast to the first-year students they surveyed, Finn *et al.* found that third-year student usage of lecture handouts or video recordings of lectures fell to 0% and 1.7%, respectively, when preparing for USMLE® Step 1 ^20^. On the other hand, they saw a staggering increase in the use of certain third-party resources, particularly UWorld® (an online question bank geared for medical board examinations), whose regular use rose from 1.9% among first-year students to 96.6% among third-year students preparing for USMLE® Step 1 ^20^. This observation has been corroborated by another group who found near universal use (99.3%) of UWorld® and First Aid™ during Step 1 preparation, while only 17.9% of the students used lecture notes or video recordings for board preparation ^5^.

### What motivates medical students to use third-party resources?

Several factors may motivate medical students to choose third-party resources as a supplementary tool or primary aid for exam preparation, but studies suggest that the primary factor is a perceived obligation to use third-party resources in order to perform well on board exams. Despite the move to a pass/fail format for both USMLE® Step 1 and some medical school curricula, exam performance is still an important factor in consideration for residencies, honor societies, and other future career opportunities. Data collected in 2021 from the National Resident Matching Program® cites USMLE® Step 1, USMLE® Step 2, and clerkship scores as among the top six important factors for consideration in a residency program ^23^. Students, well aware of how exam scores help them compete as an applicant, perceive third-party resources as a necessity to their exam preparation efforts ^4^. This perceived necessity permeates medical school classes as students encourage classmates to purchase and use third-party resources. For example, in-depth interviews with medical students of all years demonstrated that students seek advice from their peers about study strategies ^17^. This is supported by Finn *et al.*, who found 62% of first-year and 78.2% of third-year medical students reported that their favorite third-party resources were recommended to them by fellow medical students ^20^. Similarly, a study of 107 third- and fourth-year medical students revealed that students select resources that have already been vetted by fellow students and found to be effective in order to save valuable time and energy ^24^.

### Potential benefits of third-party resource use: are they effective for board preparation?

Most existing literature shows a positive association between consistent use of third-party resources and board scores ^4, 5, 25, 26^. For instance, only one study, to our knowledge, recorded no significant improvement in test scores after the purchase of third-party resources, but it noted use of resources may have been inconsistent between surveys taken after each of their six exams ^10^. On the other hand, other studies have shown that students who used third-party resources in their preparation performed significantly higher on exams compared to students who did not use the resources ^26– 28^. For example, one study instituted a “student-initiated curriculum,” wherein medical students were provided with First Aid™ and UWorld® by the school during second year ^26^. They found students using this curriculum had a significantly higher average Step 1 score and passing rate compared to prior cohorts not using this curriculum ^26^.

Certain third-party resources have been cited more frequently for improving board performance. Of note, UWorld® and First Aid™ have been popular among students who perform well on USMLE® Step 1. Examining eight studies mentioning student use of First Aid™ ^5, 19, 21, 22, 26, 28– 30^, Burk-Rafel *et al.* found multiple passes through First Aid™ were associated with quantifiable increases in Step 1 scores ^5^. Across all 19 included studies in Hirumi *et al.*, use of UWorld® alone or in combination with other third-party resources was correlated with better USMLE® performance ^4^.

Use of other third-party platforms, such as Pathoma®, Boards & Beyond®, Sketchy Medical®, USMLE Rx™, Anki™, Kaplan®, NBME® Comprehensive Basic Exam, and Firecracker™ have been noted for their positive associations with Step 1 scores ^4, 5, 25, 28^. One study even found greater use of a third-party Qbank equalized exam performance between students with lower MCAT® scores and those with higher MCAT® scores ^31^. Lastly, multiple third-party resources used in combination have been linked with corresponding improvements in Step 1 performance ^4^.

### Potential drawbacks of third-party resource use: do they exacerbate inequity in access to medical education?

The financial burden of third-party co-curricular resources rarely has been discussed in the literature ^4^, yet it presents a significant problem to medical students, a majority of whom graduate with educational debt averaging over $190,000 ^32^. As of 2023, online educational tools offered in parallel to the medical school curriculum are primarily subscription-based, with higher prices providing longer subscription periods or access to additional content or reset options ^33– 37^. Table 1 compares the cost of subscription packages to popular third-party educational resources designed for USMLE® Step 1 preclinical content as of July 2023 ^31^. Pricing details were obtained from the official websites of each third-party resource ^33– 40^. The rising costs of third-party resources also pose an economic challenge ^7^. Indeed, the subscription pricing model of most third-party resources may be particularly detrimental for medical students without a source of income and from a lower socioeconomic background, as studies describe a positive association between early use of third-party resources and Step 1 performance ^4^. This suggests that a longer subscription period (and higher costs) may be necessary to reap the benefit of these resources, contributing to an increased financial burden for medical education.

**Table 1.** Pricing of One-Time Purchase or Subscription Packages to Third-Party Board Preparation Resources Designed for USMLE Step 1 Curriculum as of July 2023.

Type	Resource	One-Time Purchase Price		
Online or paperback	First Aid™ for the USMLE Step 1 2023	$65 as sold by McGraw Hill® ^38^		
		1-month Access	6-month Access	12-month Access
Online only	USMLE Rx™ Qbank by First Aid ^36^	$129	$249	$299
	Boards & Beyond® ^37^	$89	$199	$249
	Pathoma® ^35^	Free trial only (provides access to 6 online videos; no textbook)	N/A (3 month for $84.95)	$99.95
	Sketchy Medical® ^33^	N/A	$299.99 (or $50/month)	$399.99 (or $33.33/ month)
	UWorld® ^34^	$319	$479	$559
	AMBOSS® Qbank ^39^ (prices include $14.99 monthly membership fee)	$163.99	$318.94	$478.88
	Osmosis® ^40^	N/A	$179	$199

This list is not exhaustive of all resources offered for Step 1 prep but rather provides a starting point to examine the costs associated with third-party resources commonly used in parallel with the Step 1 preclinical curriculum. Abbreviations: Qbank (Question bank), N/A (Not available) or not offered by the manufacturing company ^33– 40^.

Despite the common use of third-party resource by medical students, faculty at undergraduate medical institutions underestimate their importance to students, furthering the burden on students who may need institutional support to purchase study tools. For instance, Graff *et al.* found that faculty and medical students had differing perceptions regarding how students satisfied their learning needs, with students using significantly more third-party resources on average (5.9 resources) than their faculty perceived (4.7 resources) ^8^. The faculty believed textbooks to be the number one supplemental resource used by students, while students reported Sketchy Medical® as their predominantly used resource ^8^. This misconception may suggest why medical schools have not considered opportunities for the inclusion of third-party resources in tuition packages, which could mitigate short-term financial burden in students. Notably, these students also expressed a desire for greater equity in access to third-party resources, describing “a competitive disadvantage emerging as a consequence to excessive costs of ancillary materials.” ^8^ More recent data suggests a separation between institutional curricula and third-party resources, which may partly explain why institutional provisions for third-party resources are scarce ^12, 41^. A qualitative study of 58 second-year medical students from seven medical schools found evidence of an “adversarial relationship between their school and third-party resources” with some noting discouragement from faculty against using these resources ^41^. Another survey-based study of US medical students found a similar lack of recognition of the merits of third-party resources and the desire among students to synergize their curriculum with third-party resources ^41^. Both studies implore institutions to investigate options to offset or alleviate the cost on medical students from third-party resources ^12, 41^. Indeed, some schools may cover the cost of third-party resources within tuition packages; however, it remains to be understood whether this truly alleviates the burden on medical students. Also, though some medical schools may provide no-cost access to one or two of these tools (i.e., a free 12-month subscription to the USMLE Rx™ question bank by First Aid), such provisions are for a limited time and may need to be renewed at a cost to achieve the benefit associated with long-term use ^26, 31^.

Barriers to equitable medical education do not start with the inequitable use of costly third-party resources; rather, they are first associated with the socioeconomic barriers to medical school admission. For instance, Australian students of lower socioeconomic status (SES) and those who were the first in their family to enter university were at a greater risk of not achieving the high level of academic achievement needed for medical school admission ^42^. This may be, in part, due to the increased commercialization of resources associated with better performance (i.e., test preparation resources, tutoring, career advising) ^43, 44^. Those of low SES who are determined to pursue a medical career regardless of barriers are faced with less social support from mentors, resulting in lower self-confidence and belief in their ability to succeed in medical school ^42, 45^. Moreover, programs to increase representation of students from under-resourced backgrounds, though helpful, have not been sufficient to equilibrate access to higher-level education ^45, 46^.

Such disparities in access are further perpetuated in medical school, where the pressure to succeed and overwhelming workload are compounded by tuition costs; USMLE® Step 1 and Step 2 examination costs; and other financial obligations ^45, 47^. In fact, a team from the University of California Davis School of Medicine found that medical students who identified as socioeconomically disadvantaged had worse scores on USMLE® Step 1 exams and fewer clerkship Honors compared to their non-socioeconomically disadvantaged counterparts ^48^. This is particularly concerning as it has been demonstrated that students of low SES backgrounds have significantly more positive attitudes toward patient-centered care ^49^. Ensuring the recruitment and academic success of medical students from varying socioeconomic backgrounds, therefore, is vital for sustaining high-quality, patient-centered care. Ensuring the equitable access of resources in medical school may thus represent one method to support the next generation of patient-focused physicians.

## Limitations and next steps

Though we have arrived at several interesting observations based on our preliminary appraisal of the literature on third-party resources, our study is limited by the fact that it neither a scoping nor a systematic review. Our study assumed that preclinical students use third-party resources. As second-year medical students, we were genuinely interested in examining the literature to help us generate hypotheses to inform research questions for a future, more robust review incorporating best practices for conducting scoping reviews. Our next step is to use this information to develop a protocol for a scoping review that will examine the financial implications and disparities in access perpetuated by the use of third-party resources outside of the traditional medical school curriculum. We are also interested in investigating the relationship between multimodal use of third-party resources (e.g., video-based resource and question bank versus question bank alone) and student performance on board examinations or a survey assessing student attitudes toward successes and failures in using third-party resources. We also acknowledge that our views as preclinical medical students predispose us to a set of unique biases that inform our appraisal of the literature. We understand our position may makes us vulnerable to certain viewpoints; therefore, future studies should consist of a more diverse group of reviewers, including pre-clinical and clinical medical students, faculty, and medical school administrators. Lastly, this study is subject to publication bias as only peer-reviewed publications were evaluated.

## Conclusions

Given the increasing use, perceived necessity, and efficacy of third-party resources for preclinical medical education, institutions should address the potentially negative consequences of their use, such as their propensity to perpetuate existing socioeconomic disparities among medical students. Medical schools must evaluate the trends of third-party resource use among their medical student population, provide structures to guide its use as co-curricular and extracurricular tools, and pursue long-term solutions to bridge the gaps in accessing these resources.

## Data Availability

No data are associated with this article.
