# Supplementary material for: The Double-Edged Sword of Third-Party Resources: Examining Use and Financial Burden of Extracurricular Tools in Medical Students
Source: MedEdPublish (2016). 2025 Jan 2;14:4. Originally published 2024 Feb 12. [Version 2] doi: 10.12688/mep.20120.2 (PMC11724203; doi:10.12688/mep.20120.2)
Supplement: Peer review history

# Reviewer report: Reviewer response for version 2

**DOI:** 10.21956/mep.22322.r40931

**Published:** 2025-02-14

**Authors:** Andrew PJ Olson

**Affiliations:** 1 University of Minnesota Medical School, Minneapolis, USA

Thank you for the opportunity to review this manuscript, which is a perspective piece, based on existing literature, about the use of third-party resources in medical education.  The discussion of the literature is generally appropriate and the conclusions are reasonable, especially the need to formally evaluate the potential positive and negative consequences of their use.  Further, the call for further reviews is appropriate but should be strengthened to call for better empirical research that seeks to formally evaluate structures and strategies for incorporating such resources into medical education (including randomized controlled trials, etc).

 A bit more meta-level discussion might also be warranted and help frame this discussion.  The phenomenon about third party resource use is described well, but the "why" behind their use could be expanded further.   What factors are leading to the use of these tools, and what factors make medical schools view them often negatively?   For example, individual faculty who receive salary support to teach could view their use as an existential threat and curriculum leaders could view their use as potentially obviating their well-designed curriculum, when in fact a more fulsome and collaborative approach is likely helpful.

 Finally, while the focus on inequity is valuable, it also might be helpful to touch on the effect and burden on students of having to complete two parallel curricula instead of one.

 Thank you for this great work, and for the opportunity to review.

Is the review written in accessible language?

Yes

Are all factual statements correct and adequately supported by citations?

Partly

Are the conclusions drawn appropriate in the context of the current research literature?

Yes

Is the topic of the review discussed comprehensively in the context of the current literature?

Partly

Reviewer Expertise:

Clinical reasoning, medical education outcomes research.

I confirm that I have read this submission and believe that I have an appropriate level of expertise to confirm that it is of an acceptable scientific standard, however I have significant reservations, as outlined above.

# Reviewer report: Reviewer response for version 2

**DOI:** 10.21956/mep.22322.r40880

**Published:** 2025-01-21

**Authors:** Silvia Lizett Olivares

**Affiliations:** 1 Tecnologico de Monterrey, Monterrey, Nuevo Leon, Mexico

There is no methodological support for "a high-level review of the literature" instead of a systematic review. The article represents a "perspective" essay which includes a critical judgment of the authors' experiences. The paper should include more references for the argumentation of the method section.

 There was no rigorous approach for searching terms since there were no inclusion or exclusion criteria. There should be more information about the searching process. 

 The conclusions are inferred from other expenses than third-party resources. It is important to address if students have replaced books and other physical materials with these resources.

Is the review written in accessible language?

Yes

Are all factual statements correct and adequately supported by citations?

Partly

Are the conclusions drawn appropriate in the context of the current research literature?

Partly

Is the topic of the review discussed comprehensively in the context of the current literature?

Yes

Reviewer Expertise:

Curriculum Design, Interprofesional education.

I confirm that I have read this submission and believe that I have an appropriate level of expertise to confirm that it is of an acceptable scientific standard, however I have significant reservations, as outlined above.

# Reviewer report: Reviewer response for version 2

**DOI:** 10.21956/mep.22322.r40785

**Published:** 2025-01-10

**Authors:** Yasar Ahmed

**Affiliations:** 1 St.Vincent's University Hospital, Dublin, Ireland

I have no further comments to make.

Is the review written in accessible language?

Yes

Are all factual statements correct and adequately supported by citations?

Partly

Are the conclusions drawn appropriate in the context of the current research literature?

Partly

Is the topic of the review discussed comprehensively in the context of the current literature?

Yes

Reviewer Expertise:

Medical Education , Educational Technology,Economics of Education and Research Methodology:

I confirm that I have read this submission and believe that I have an appropriate level of expertise to confirm that it is of an acceptable scientific standard.

# Reviewer report: Reviewer response for version 1

**DOI:** 10.21956/mep.21545.r36554

**Published:** 2024-05-06

**Authors:** Yasar Ahmed

**Affiliations:** 1 St.Vincent's University Hospital, Dublin, Ireland

The article states that the use of third-party resources has increased significantly since before the COVID-19 pandemic. This claim should be supported by studies or data evidencing this trend, preferably with citations that track the use over time.

 The assertion that these resources have rarely been evaluated needs a citation to studies that have attempted such evaluations, or a statement should be provided indicating a lack of existing literature.

 The methodological approach involving the review of 31 peer-reviewed articles needs details about the criteria for article selection and the relevance of these articles to the topic. The claim here appears sound but needs backing through a description of the selection process or a reference to a methodological appendix

 Claims about the task-dependent use of resources and their roles in different phases of medical education should be backed by specific studies or surveys that have observed these trends.

 The financial impact mentioned, including the range of subscription costs from $100 to $479, should directly reference the sources of these pricing details or market surveys

 The conclusions highlight that third-party resources, while beneficial in improving board exam scores, contribute to financial strain and may exacerbate socioeconomic disparities among medical students

 The article successfully supports these conclusions through: 

 1- **Efficacy Data**: 

 2- **Financial Strain**:

 3- **Socioeconomic Disparities**

 The conclusions could be strengthened by a discussion of any potential biases in the sources of data, such as the possibility of publication bias in studies demonstrating the efficacy of third-party resources.

Is the review written in accessible language?

Yes

Are all factual statements correct and adequately supported by citations?

Partly

Are the conclusions drawn appropriate in the context of the current research literature?

Partly

Is the topic of the review discussed comprehensively in the context of the current literature?

Yes

Reviewer Expertise:

Medical Education , Educational Technology,Economics of Education and Research Methodology:

I confirm that I have read this submission and believe that I have an appropriate level of expertise to confirm that it is of an acceptable scientific standard, however I have significant reservations, as outlined above.

# Response

**Published:** 2024-12-14

**Authors:** Dimitrios Papanagnou

**Affiliations:** Emergency Medicine, Thomas Jefferson University, Philadelphia, Pennsylvania, USA

**Author Response:**

**Reviewer #3: The article states that the use of third-party resources has increased significantly since before the COVID-19 pandemic. This claim should be supported by studies or data evidencing this trend, preferably with citations that track the use over time.**  

**Authors’ Response: **Thank you for this comment. We added data evidencing the trend that third-party resource use has increased significantly since the COVID-19 pandemic. We reworded the second sentence to better explain what third-party resources are and removed the original statement for more clarity. We ensured all claims were appropriately supported with citations in the paper as per the MedEd Publish guidelines for reporting.

- Abstract: “ *According to the Association of American Medical Colleges (AAMC) Year 2 questionnaire, the percentage of students using online medical education videos (Boards and Beyond* *Ò,* *Sketchy Medical* *Ò* *, Youtube) at least once per week increased from 47.7% in 2015 to 70.1% in 2022.* *The transition to virtual learning in 2020 fostered a greater reliance on these mostly online third-party resources (or educational tools separate from the in-house medical curriculum) resources during medical training, yet their contributions have rarely been evaluated*.”

**Reviewer's comment:** **1. The assertion that these resources have rarely been evaluated needs a citation to studies that have attempted such evaluations, or a statement should be provided indicating a lack of existing literature.**  

**Authors’ Response:** Thank you for this comment. MedEd Publish prohibits adding citations in the abstract, so we included a statement indicating the lack of existing literature.

- **Abstract:** “… *yet existing literature have rarely evaluated their contributions*.”

**Reviewer's comment: 2. The methodological approach involving the review of 31 peer-reviewed articles needs details about the criteria for article selection and the relevance of these articles to the topic. The claim here appears sound but needs backing through a description of the selection process or a reference to a methodological appendix.**  

**Authors’ Response:** Thank you for this comment. Similar concerns were brought up by Reviewer #1 and Reviewer #2, so we adjusted this accordingly.

**Reviewer's comment: 3. Claims about the task-dependent use of resources and their roles in different phases of medical education should be backed by specific studies or surveys that have observed these trends.**  

**Authors’ Response: **We agree that the use of the word “task-dependent” brought confusion. We adjusted this statement in response to Reviewer #2’s suggestion and ensured all studies were cited accordingly. We added additional citations as per Reviewer #3’s suggestion.

- “ *Medical students seek out and use a variety of third-party resources that aid their goals, regardless of the quantity. ^4,10,12,13^* ^”^

**Reviewer's comment:** **4. The financial impact mentioned, including the range of subscription costs from $100 to $479, should directly reference the sources of these pricing details or market surveys**  

**Authors’ Response: **Thank you for this comment. We have included a statement clarifying the sources of the pricing details in Table 1 and ensured all prices were appropriately cited in the manuscript, table, and table caption.

- “ *Pricing details were obtained from the official websites of each third-party resource. ^33–40^* ^”^

**Reviewer's comment:** **5. The conclusions highlight that third-party resources, while beneficial in improving board exam scores, contribute to financial strain and may exacerbate socioeconomic disparities among medical students. The article successfully supports these conclusions through:** **1-Efficacy Data: 2-Financial Strain: 3-Socioeconomic Disparities. The conclusions could be strengthened by a discussion of any potential biases in the sources of data, such as the possibility of publication bias in studies demonstrating the efficacy of third-party resources.**  

**Authors’ Response: **Thank you for your thorough review of our work. We included statements related to publication bias and other biases that may have arisen in review of this data in the newly added Limitations and Next Steps section.

- Limitations and Next Steps: “ *Also, we acknowledge that our perspective as preclinical medical students predispose us to a set of biases that may have informed our appraisal of the literature. As we are students evaluating resources made for students, we understand our position may have made us vulnerable to certain perspectives, so future studies should aim to have a more balanced group of reviewers including pre-clinical and clinical medical students, faculty, and medical school administration members. Lastly, this study is subject to publication bias as only peer-reviewed publications were evaluated*.”

# Reviewer report: Reviewer response for version 1

**DOI:** 10.21956/mep.21545.r36553

**Published:** 2024-05-03

**Authors:** Katherine S. McOwen

**Affiliations:** 1 Association of American Medical Colleges, Columbia, USA

Thank you for your exploration of the use of third-party resources, a timely topic.  I found your review of the literature to be helpful, but your structure was a bit difficult to follow. I offer suggestions that might enhance the reader’s experience of your work.

**Abstract**

 Please align your abstract more closely with your work, for example is the purpose of this work to look specifically at cost of resources, or at equitable access of resources?

**Background**

 This works includes a helpful introduction to the use of third-party resources in medical education.

 Paragraph 2 – I’m not sure I understand the impact of the COVID pandemic on the use of third-party resources, since 90% of students were using them before the pandemic.  Do you mean that even greater than 90% of students were using them after the pandemic, or that their use became more varied or different in some way during and after the pandemic?

 Paragraph 4 – thank you for clearly stating the purpose of this work, an exploration of the use of third-party resources and the potential for increased inequity between students because of their expense.

**Methods**

 The methods section requires additional information to assist the reader in understanding what you are doing. Describe your search strategy, the time you did the search, what languages you included, and your inclusion/exclusion criteria.  There is not enough detail in your work for the reader to understand the context for your findings.

 While I find the use of questions as paragraph headers useful for the reader in the results section, include the questions as well as your process for identifying the questions in your methods section so the reader can follow your logic and knows what to expect in the results.

**Results**

 Label your results section.

 In the paragraph that follows, “what types of third-party resources are used by medical students”, the first sentence feels out of place and requires some additional context to assist the reader in understanding what you are saying. The entire paragraph is difficult to follow, I suggest re-writing to answer the question more directly.

 It would be helpful to direct that with your thoughts about where future research might focus to address some of the questions that remain after your review of the literature. For example, I am curious about the relationship between third-party resources and student self-directed learning, potentially levelling the playing field between students with different resources or preparation.

Is the review written in accessible language?

Yes

Are all factual statements correct and adequately supported by citations?

Yes

Are the conclusions drawn appropriate in the context of the current research literature?

Yes

Is the topic of the review discussed comprehensively in the context of the current literature?

Partly

Reviewer Expertise:

medical education, qualitative methods

I confirm that I have read this submission and believe that I have an appropriate level of expertise to confirm that it is of an acceptable scientific standard, however I have significant reservations, as outlined above.

# Response

**Published:** 2024-12-13

**Authors:** Dimitrios Papanagnou

**Affiliations:** Emergency Medicine, Thomas Jefferson University, Philadelphia, Pennsylvania, USA

**Author Response:**

**Reviewer #2: Thank you for your exploration of the use of third-party resources, a timely topic.  I found your review of the literature to be helpful, but your structure was a bit difficult to follow. I offer suggestions that might enhance the reader’s experience of your work.**  

**Authors’ Response:** Thank you for your thorough review of our work.  

**Reviewer's comment: 1. Abstract - Please align your abstract more closely with your work, for example is the purpose of this work to look specifically at cost of resources, or at equitable access of resources?**  

**Authors’ Response: **A similar concern was brought up by Reviewer #1, so we revised the aim statement to make it more specific. We adjusted the statement to specify that we examined the cost of third-party resources to explain how it may perpetuate existing barriers to equitable medical education.

- “ *As second-year medical students steeped in this landscape, we aimed to review the usage, efficacy, and drawbacks of third-party resources and their implications for medical education, particularly their costs or financial burden on students and how it may perpetuate existing barriers to equitable medical education.”*

**Reviewer's comment:** **2. Background - This work includes a helpful introduction to the use of third-party resources in medical education. Paragraph 2 – I’m not sure I understand the impact of the COVID pandemic on the use of third-party resources, since 90% of students were using them before the pandemic.  Do you mean that even greater than 90% of students were using them after the pandemic, or that their use became more varied or different in some way during and after the pandemic?**  

**Authors’ Response: **Thank you for this insightful comment. We agree that the verbiage confused the original intention of this sentence, which is that the transition to virtual learning during the COVID-19 pandemic led to an increase in the use of third-party resources as mentioned in a later sentence. We removed the confusing wording to make this clear.

- “ *This expanding supply of third-party resources may arise from an increased demand from medical students which was amplified by the COVID-19 pandemic*.”

**Reviewer's comment:** **3. Background - Paragraph 4 – thank you for clearly stating the purpose of this work, an exploration of the use of third-party resources and the potential for increased inequity between students because of their expense.**  

**Authors’ Response: **Thank you for this comment.  

**Reviewer's comment: 4. Methods - The methods section requires additional information to assist the reader in understanding what you are doing. Describe your search strategy, the time you did the search, what languages you included, and your inclusion/exclusion criteria.  There is not enough detail in your work for the reader to understand the context for your findings.** **While I find the use of questions as paragraph headers useful for the reader in the results section, include the questions as well as your process for identifying the questions in your methods section so the reader can follow your logic and knows what to expect in the results.**  

**Authors’ Response: **Thank you for this comment. Reviewer #1 brought up a similar concern in reporting the methods (item 3), so this was addressed as stated above. We also added statements in the Methods (now Literature Search) section to follow the Reviewer’s recommendation. Also, we reframed the final paragraph header to be a question to follow parallel structure.

- Literature Search: “ *After common ideas were collected, key findings were designed as questions to lead each paragraph: “What types of third-party resources are used by medical students?”, “How do medical students incorporate third-party resources in their education?”, “What motivates medical students to use third-party resources?”, “Potential benefits of third-party resource use: are they effective for board preparation?”. “Potential drawbacks of third-party resource use: do they increase inequity in access to medical education?*”.”
- “ *Potential drawbacks of third-party resource use: do they increase inequity in access to medical education?*”

**Reviewer's comment:** **5. Results - Label your results section. In the paragraph that follows, “what types of third-party resources are used by medical students”, the first sentence feels out of place and requires some additional context to assist the reader in understanding what you are saying. The entire paragraph is difficult to follow, I suggest re-writing to answer the question more directly.**  

**Authors’ Response:** Thank you for this comment. We agree that the use of the word “task-dependent” raises confusion, so we removed this statement entirely. We changed the first sentence, so it answered the question more directly. We also ensured all mentions of the types of third-party resources used were cited appropriately. Lastly, we organized this paragraph better with transitions for clarity.

- “ *Medical students seek out and use a variety of third-party resources that aid their goals, regardless of the quantity* *. For instance, researchers at the Kirk Kerkorian School of Medicine conducted post-exam surveys on medical students taking National Board of Medical Education (NBME)-based exams bi-weekly and found that more than half of respondents (55%) used between six and eight resources for exam preparation. ^10^ Among the most used resources were Boards & Beyond (video lectures with post-video quizzes), First Aid (exam review book), and Kaplan (practice question bank) in descending order. ^10^ This may be partly explained by students’ desire to seek out third-party resources that are concise, easy to use, and that support long-term retention and recall. ^4,8,13^*
- *Additionally, Hirumi et al. discusses a strong preference by medical students for resources that offer practice questions (e.g., Boards & Beyond, Kaplan, UWorld, USMLE Rx by First Aid, Sketchy Medical) as they allow students to gain comfort with a format similar to that of the national board examinations. ^4^ Such question banks (or Qbanks) provide an opportunity to practice well-supported educational techniques like spaced retrieval and active recall. ^4,14,15^ Indeed, students also seek resources that cover “high-yield” topics, or content that has been regularly tested by board examiners and is considered essential content to review before examinations. ^13,16^ This may further motivate students to use third-party Qbanks, as practice questions generally emphasize “high-yield” content. ^4,17,18^* ^”^

**Reviewer's comment:** **6. Results - It would be helpful to direct that with your thoughts about where future research might focus to address some of the questions that remain after your review of the literature. For example, I am curious about the relationship between third-party resources and student self-directed learning, potentially levelling the playing field between students with different resources or preparation.**

**Authors’ Response:** Thank you for this valuable comment. We have included statements to our newly added Limitations section to address future studies. We changed the title of the Limitations section to Limitations and Next Steps to highlight this.

- Limitations and Next Steps: “ *Next steps would involve a systematic review assessing the financial implications and disparities in access perpetuated by the use of third-party resources outside of the traditional medical school curriculum. Future studies may also involve investigating the relationship between multimodal use of third-party resources (i.e., video-based resource and question bank versus question bank alone) and student performance on board examinations or a survey assessing student attitudes toward successes and failures in using third-party resources*.”
- “… *so future studies should aim to have a more balanced group of reviewers including pre-clinical and clinical medical students, faculty, and medical school administration members*.”

# Reviewer report: Reviewer response for version 1

**DOI:** 10.21956/mep.21545.r36094

**Published:** 2024-02-29

**Authors:** Gary Beck Dallaghan

**Affiliations:** 1 University of Texas Health Science Center, Tyler, USA

Thank you for submitting this article on third-party medical student resource usage and costs.  It is a topic I have been interested in for many years.  The comments I am making will hopefully lead to improvements to what you have submitted.

 First and foremost, although you have indicated this is more of a scholarly perspective, you have used a review of the literature to enhance your argument.  In my experience writing pieces like this, it was beneficial to use guidelines for reporting narrative reviews of the literature to get the work published.  It also helps to be more comprehensive in your reporting.  I would suggest reading and following guidelines laid out in the following article: (Ref [1])

 Abstract:

 The aim of the study could be improved. By that I mean it was not specific, particularly in light of what you report.  You might want to revise it.

 The Methods of your abstract are not methods. I want to know what databases you searched. What search terms were used? What was the timeframe of the search? Were references mined for additional articles?  What you have in the Methods belongs in the Results.

 You need to spell out USMLE when you use it the first time.  For international journals, they are not going to know what that is.

 In the Results, beginning with "...suggesting that third-party resource use..." to the end of the paragraph belongs in your Conclusion section.  Those are your interpretations which is where they belong.

 The Methods section needs more details as I noted above.  I think if you read the Ferrari article you will know how to better structure this section.

 Since you are using a Results section in your abstract, you really should use that in the manuscript. Instead, we went from a single paragraph of Methods to results with the first subheading.  It was jarring.  The Results also need to inform the reader how many articles you found in total and if any were removed due to not including information you wanted.  

 The second category of the results should start with a sentence that is structured differently from the first section.  Those two sentences read so closely I had to look up to make sure I was reading something different.

 One thing I am wondering if you are able to draw out in the Discussion is whether or not medical schools have reported they pay for third-party resources.  I know students use them, but I also know schools (mine included) pays for the subscription.  So if that is the case, is it really a financial burden on the student?  Even if you cannot find the data, it may be worth exploring that possibility as part of the discussion.

Is the review written in accessible language?

Yes

Are all factual statements correct and adequately supported by citations?

Yes

Are the conclusions drawn appropriate in the context of the current research literature?

Partly

Is the topic of the review discussed comprehensively in the context of the current literature?

Partly

Reviewer Expertise:

medical student education, qualitative research, literature reviews, quantitative research

I confirm that I have read this submission and believe that I have an appropriate level of expertise to confirm that it is of an acceptable scientific standard, however I have significant reservations, as outlined above.

## References

[1] : Writing narrative style literature reviews. *Medical Writing* .2015;24(4) : 10.1179/2047480615Z.000000000329 230-235 10.1179/2047480615Z.000000000329

# Response

**Published:** 2024-12-13

**Authors:** Dimitrios Papanagnou

**Affiliations:** Emergency Medicine, Thomas Jefferson University, Philadelphia, Pennsylvania, USA

**Author Replies:**

**Reviewer #1: Thank you for submitting this article on third-party medical student resource usage and costs.  It is a topic I have been interested in for many years.  The comments I am making will hopefully lead to improvements to what you have submitted.**  

**Authors’ Response: **Thank you for your thorough review of our work.  

**1. First and foremost, although you have indicated this is more of a scholarly perspective, you have used a review of the literature to enhance your argument.  In my experience writing pieces like this, it was beneficial to use guidelines for reporting narrative reviews of the literature to get the work published.  It also helps to be more comprehensive in your reporting.  I would suggest reading and following guidelines laid out in the following article: (Ref [1])**  

**Authors’ Response: **Thank you for sharing this article. We have reviewed the article in detail and though our study methods aligned with some of the guidelines mentioned in the article (i.e., multiple independent reviewers, search terms, critical assessment of the literature extracted), our paper was intended to serve as a commentary or perspective. Our goal was to inform a hypothesis that can fine-tune research questions to for a formal, more robust review, which is now described in our newly-added Limitations section. We have included additional language explaining our approach in the revised Methods (please see ‘Literature Search’ sub-section). We have also included a Limitations section to expand upon the following concerns:

- **Background: **changed “ *argue*” to *“hypothesize*”
- “ *..queried PubMed database using search terms…during June to July 2023*”
- “ *No inclusion or exclusion criteria was implemented besides only reviewing articles in English. Some references were mined for additional results as per the discretion of each reviewer.”*
- “ *Each study was appraised for key endpoints and conclusions, methods used to reach the initial hypothesis, strengths, limitations, and future directions suggested by the article.”*
- **Limitations: **“ *Though we have arrived at interesting findings upon appraisal of the literature on third-party resources, this is limited by the fact that this is not a systematic or scoping review. This study was produced upon the assumption that preclinical students were using third-party resources. As second-year medical students ourselves, we wanted to explore the literature to inform preliminary research questions for future studies. Next steps will involve a scoping review assessing the financial implications and disparities in access perpetuated by the use of third-party resources outside of the traditional medical school curriculum. We also acknowledge that our perspective as preclinical medical students predisposes us to a set of biases that may have informed our appraisal of the literature and our hypothesis generation. Future studies should aim to have a more balanced group of reviewers including pre-clinical and clinical medical students, faculty, and medical school administration members. Lastly, this study is subject to publication bias as only peer-reviewed publications were evaluated.”*

 
**2. Abstract: The aim of the study could be improved. By that I mean it was not specific, particularly in light of what you report.  You might want to revise it.**  

**Authors’ Response:** We have made the aim more specific in accordance with the Reviewer’s recommendation and the subtopics of our Results section.

- “ *As second-year medical students steeped in this landscape, we aimed to review the usage, efficacy, and drawbacks of third-party resources and their implications for medical education, particularly their financial burden on students.”*

**3. The Methods of your abstract are not methods. I want to know what databases you searched. What search terms were used? What was the timeframe of the search? Were references mined for additional articles?  What you have in the Methods belongs in the Results.**  

**Authors’ Response:** We have added statements in the Methods of the abstract and adjusted the placement of prior statements as per the Reviewer’s recommendation.

- **Methods:** “ *Four authors searched the PubMed database from June to July 2023 using* *the search terms “prevalence,” “efficacy,” and “disparities introduced by third-party board preparation resources.” All peer-reviewed articles in English were evaluated, and no inclusion or exclusion criteria were implemented. Some references were mined for additional results as per the discretion of the reviewer.”*
- **Results:** “ *31 studies were examined for common themes and findings to provide a snapshot of the existing literature on third-party resources for medical students and their equitable access.”*

 
**4. You need to spell out USMLE when you use it the first time.  For international journals, they are not going to know what that is.**  

**Authors’ Response: **This has been adjusted in the abstract and manuscript.

**5. In the Results, beginning with "...suggesting that third-party resource use..." to the end of the paragraph belongs in your Conclusion section.  Those are your interpretations which is where they belong.**  

**Authors’ Response:** Thank you for this valuable comment. This has been adjusted in the abstract.

**6. The Methods section needs more details as I noted above.  I think if you read the Ferrari article you will know how to better structure this section.**  

**Authors’ Response:** This has been corrected as stated above.  

**7. Since you are using a Results section in your abstract, you really should use that in the manuscript. Instead, we went from a single paragraph of Methods to results with the first subheading.  It was jarring.  The Results also need to inform the reader how many articles you found in total and if any were removed due to not including information you wanted.**  

**Authors’ Response: **Thank you for this thoughtful comment. Since we used a conceptual framework (discussed by themes) to organize our results, we have added a “Results” section header following our Methods (now Literature Search) section. We renamed the Methods section to better capture what is discussed in the section. We also addressed number of articles found and inclusion/exclusion criteria as per the Reviewer’s recommendation.  

**8. The second category of the results should start with a sentence that is structured differently from the first section.  Those two sentences read so closely I had to look up to make sure I was reading something different.**  

**Authors’ Response:** Thank you for this comment. We renamed the heading of our second subsection in the Results to “ *How do medical students incorporate third-party resources in their education?”.*  

**9. One thing I am wondering if you are able to draw out in the Discussion is whether or not medical schools have reported they pay for third-party resources.  I know students use them, but I also know schools (mine included) pays for the subscription.  So if that is the case, is it really a financial burden on the student?  Even if you cannot find the data, it may be worth exploring that possibility as part of the discussion.**  

**Authors’ Response: **Thank you for this insightful query. In searching for data to address this comment, we found recently published studies that further expand our discussion of the “adversarial relation between medical schools and third-party resources.” We have added this and additional statements to address the Reviewer’s query.

- “ *More recent data suggests a separation between institutional curricula and third-party resources, which may partly explain why institutional provisions for third-party resources are scarce. ^41,42^ A qualitative study of 58 second-year medical students from seven medical schools found evidence of an “adversarial relationship between their school and third-party resources” with some noting discouragement from faculty against using these resources. ^42^ Another survey-based study of US medical students found a similar lack of recognition of the merits of third-party resources and the desire among students to synergize their curriculum with third-party resources. ^42^”*
- *“Both studies implore institutions to investigate options to offset or alleviate the cost on medical students from third-party resources. ^41,42^”*
- *“Indeed, some schools may cover the cost of third-party resources within tuition packages; however, it remains to be understood whether this truly alleviates the burden on medical students*.”
